# Whole-mol­ecule disorder of the Schiff base compound 4-chloro-*N*-(4-nitro­benzyl­idene)aniline: crystal structure and Hirshfeld surface analysis

**DOI:** 10.1107/S2056989020002212

**Published:** 2020-02-18

**Authors:** Sundararaman Leela, Ashokkumar Subashini, Philip Reji, Kandasami Ramamurthi, Helen Stoeckli-Evans

**Affiliations:** a Department of Physics, Ethiraj College for Women, Chennai - 600 008, Tamilnadu, India; b Crystal Growth and Thin Film Laboratory, School of Physics, Bharathidasan University, Tiruchirappalli - 620 024, India; c PG and Research Department of Physics, Srimad Andavan Arts and Science College, Tiruchirappalli - 620 005, India; d Light and Matter Physics Group, Raman Research Institute, C. V. Raman Avenue, Sadashivanaga, Bangalore - 560 080, India; e Department of Bio-Medical Engineering, Aarupadai Veedu Institute of Technology, Vinayaga Mission’s Research Foundation, Vinayaga Nagar, Paiyanoor, 603 104, Tamil Nadu, India; f Institute of Physics, University of Neuchâtel, rue Emile-Argand 11, CH-2000 Neuchâtel, Switzerland

**Keywords:** crystal structure, Schiff bases, non-linear optical properties, disorder, hydrogen bonding, Hirshfeld surface analysis

## Abstract

In the crystal of the title Schiff base compound, C_13_H_9_ClN_2_O_2_, [CNBA; systematic name: (*E*)-*N*-(4-chloro­phen­yl)-1-(4-nitro­phen­yl)methanimine], the CNBA mol­ecule shows whole-mol­ecule disorder (occupancy ratio 0.65:0.35), with the disorder components related by a twofold rotation about the shorter axis of the mol­ecule. The aromatic rings are inclined to each other by 39.3 (5)° in the major component and by 35.7 (9)° in the minor component. In the crystal, C—H⋯O hydrogen bonds predominate in linking the major components, while weak C—H⋯Cl inter­actions predominate in linking the minor components. The result is the formation of corrugated layers lying parallel to the *ac* plane. The crystal packing was analysed using Hirshfeld surface analysis and compared with related structures.

## Abstract 2

In the solid state the title compound shows full-mol­ecule disorder (occupancy ratio 0.65: 0.35), generated by a twofold rotation about the shorter axis of the mol­ecule.

## Chemical context   

A number of benzyl­ideneaniline derivatives crystallize in non-centrosymmetric space groups and are therefore of inter­est for their non-linear optical properties (Bar & Bernstein, 1977 ▸; Batra *et al.*, 2004 ▸). In 1970, Bürgi & Dunitz (1970 ▸) analysed a number of *N*-benzyl­anilines and found that they were twisted about the N=C bond unlike *trans*-stilbenes (see for example: Behrnd *et al.*, 2010 ▸; De Borger *et al.*, 2005 ▸) or *trans*-azo­benzenes (see for example: Huang *et al.*, 2002 ▸; Bushuyev *et al.*, 2016 ▸), which are almost planar.
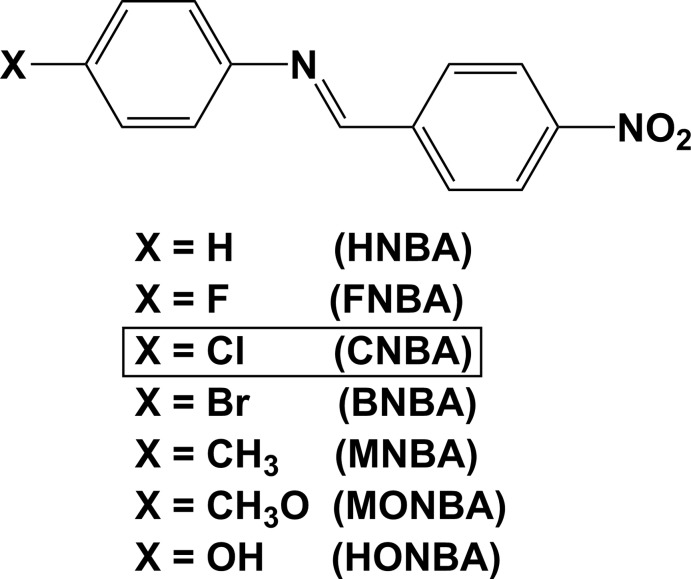


Benzyl­ideneaniline derivatives are known to exhibit disorder and Bernstein and collaborators (Bar & Bernstein, 1983 ▸; Kluge *et al.*, 2003 ▸) have defined the different types of orientational disorder of these compounds, where the mol­ecules may be oriented in different ways but in the two or more dispositions each atom is essentially superimposed on another at any one crystallographic site. Static disorder around the C=N bond is also responsible for the apparent shortening of the C=N bond at room temperature (Bar & Bernstein, 1984 ▸). This phenomenon has also been studied by Harada *et al.* (2004*a* ▸), who, by means of a variable temperature study, concluded that the shortening depends on temperature and is due to a torsional vibration of the C–phenyl and N–phenyl bonds in the crystals.

**Figure 1:**
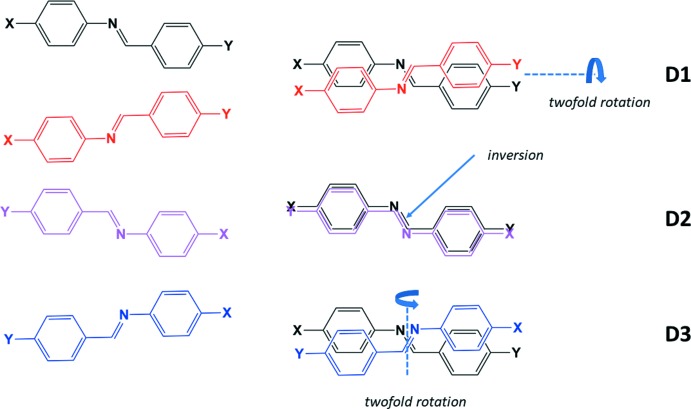
Disorder types in benzyl­ideneanilines.

The crystal structures of a number of disordered benzyl­ideneaniline compounds have been reported on and various forms of the disorder have been analysed (Bar & Bernstein, 1977 ▸, 1984 ▸; Harada *et al.*, 2004*a* ▸,*b* ▸). The disorder appears to fall into three categories (Fig. 1 ▸): *D*1 – twofold rotation about the longer axis of the mol­ecule, *D*2 – the mol­ecule is located about a crystallographic center of symmetry, and *D*3 – twofold rotation about the shorter axis of the mol­ecule.

Type *D*1 disorder has been observed for one of the two independent mol­ecules in the crystal of *N*-(4-nitro­benzyl­idene)aniline at 300 and 200 K, but the disorder is not present at 90 K (Harada *et al.*, 2004*b* ▸). Orientational disorder about a center of symmetry (type *D*2) was found in *N*-(*p*-chloro­benzyl­idene)-*p*-chloro­aniline (Bar & Bernstein, 1982 ▸; Bernstein & Schmidt, 1972 ▸). Type *D*3 disorder has been observed for *N*-benzyl­ideneaniline (Bernstein & Izak, 1976 ▸; Harada *et al.*, 2004*a* ▸) and for 4-methyl-4′-meth­oxy­benzyl­ideneaniline (Harada *et al.*, 2004*a* ▸).

Three forms of *p*-methyl-*N*-(*p*-methyl­benzyl­idene)aniline (Bernstein, Bar & Christensen, 1976 ▸; Bar & Bernstein, 1982 ▸; Bar & Bernstein, 1977 ▸) have been shown to exist: Form I (Bar & Bernstein, 1982 ▸), crystallizes in space group *P*2_1_/*c* and the C=N bond of the mol­ecule is located about a center of symmetry, hence the mol­ecule has type *D*2 disorder; form II (Bar & Bernstein, 1977 ▸) crystallizes in space group *P*2_1_ and the mol­ecule is not disordered; form III (Bar & Bernstein, 1977 ▸; Harada *et al.*, 2004*b* ▸), has a fourfold disorder with the mol­ecule being located about a center of symmetry and has a twofold rotation about the longer axis of the mol­ecule (*D*1 + *D*2).

In the past few years some benzyl­ideneaniline compounds have been synthesized using *p*-nitro­benzaldehyde as one of the reactants; for example, 4-nitro-benzyl­ideneaniline (HNBA; Harada *et al.*, 2004*b* ▸), 4-fluoro-4′-nitro-benzyl­idene­aniline (FNBA; Subashini *et al.*, 2013*b* ▸), 4-bromo-4′-nitro-benzyl­ideneaniline (BNBA; Subashini *et al.*, 2013*a* ▸) and 4-hy­droxy-4′-nitro-benzyl­ideneaniline [systematic name: 4-[(*E*)-(4-nitro­benzyl­idene)amino]­phenol] (HONBA; Atioğlu *et al.*, 2015 ▸).

To continue the series of 4-halogen species, we report herein on the crystal structure of 4-chloro-4′-nitro-benzyl­ideneaniline (CNBA). It was previously synthesized by Batra *et al.* (2004 ▸), who found that the crystals they obtained showed good second harmonic generation (SHG) of 1.064 micron wavelength radiation. The crystal structure analysis carried out for CNBA in this work shows that it crystallizes in the centrosymmetric space group *P*2_1_/*c*, and that the mol­ecule has positional disorder (type *D*3), hence no SHG properties are expected for this particular sample. It is inter­esting to note that the structure of 4-bromo-4′-nitro­benzyl­ideneaniline (BNBA) crystallizes in a non-centrosymmetric space group (*A*2), while the title compound and 4-fluoro-4′-nitro­benzyl­idene aniline (FNBA; Subashini *et al.*, 2013*b* ▸) both crystallize in space group *P*2_1_/*c*.

## Structural commentary   

**Figure 2:**
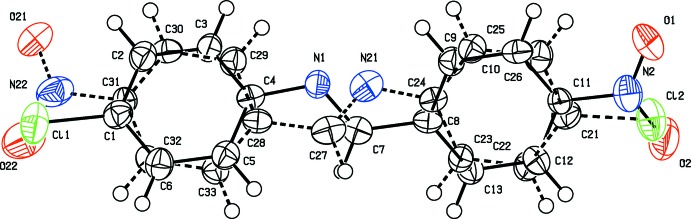
Mol­ecular structure of CNBA, with atom labelling. The displacement ellipsoids are drawn at the 50% probability level. The major component is shown with solids bonds, while the minor component is shown with dashed bonds.

**Figure 3:**
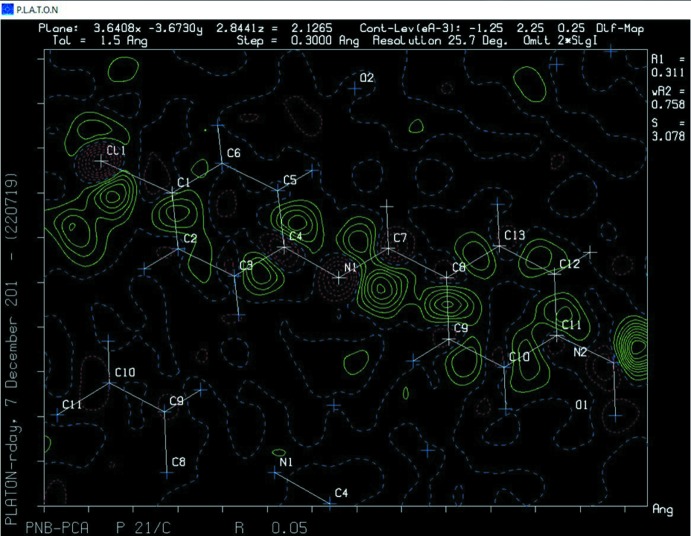
A difference electron-density map showing the density peaks related to the minor disordered component.

The mol­ecular structure of CNBA is shown in Fig. 2 ▸. It crystallizes in the centrosymmetric monoclinic space group *P*2_1_/*c*, and is disordered with a twofold rotation about the shorter axis of the mol­ecule – type *D*3. The twofold axis almost bis­ects the central C=N bond, so that the two component mol­ecules are superimposed head-to-tail, as shown clearly in a difference-Fourier map (Fig. 3 ▸). They have an occupancy ratio that, after initial refinement, was fixed at 0.649:0.351. As mentioned above, this type of disorder (*D*3) has been observed previously for related phases.

The configuration about the C=N bond is *E* in both components. The dihedral angle between the benzene rings of the major component CNBA_1 (C1–C6 and C8–C13) is 38.6 (2)°, and that between rings C21–C26 and C28–C33 of the minor component (CNBA_2) is 36.5 (4)°. In CNBA_1 the N1=C7 bond length is 1.291 (6) Å, while for CNBA_2 the equivalent N21=C27 bond length is 1.234 (12) Å. The NO_2_ group, N2/O1/O2, in CNBA_1 is inclined to benzene ring C8–C13 by 2.2 (7)°, and atom Cl1 is displaced by 0.016 (3) Å from benzene ring C1–C6. In component CNBA_2, the NO_2_ group, N22/O21/O22, is inclined to benzene ring C28–C33 by 9.0 (15)°, while atom Cl2 lies in the plane of the benzene ring C21–C26 [deviation 0.002 (5) Å].

## Supra­molecular features   

**Figure 4:**
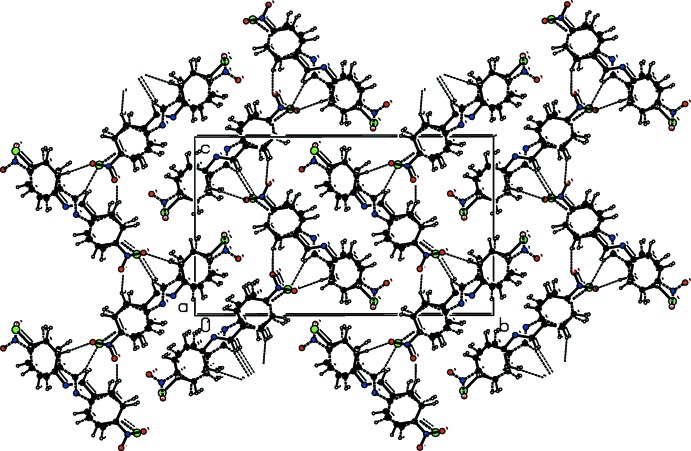
A view along the *a* axis of the crystal packing of CNBA. The hydrogen bonds (see Table 1 ▸) are shown as dashed lines.

**Figure 5:**
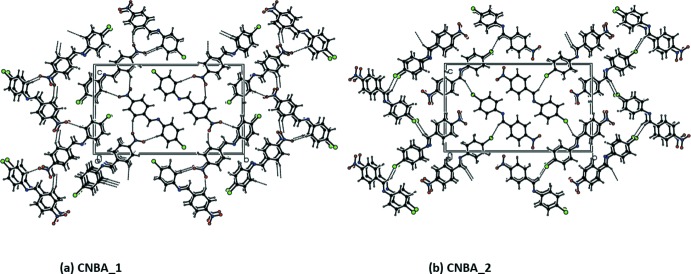
A view along the *a* axis of the crystal packing of (*a*) the major disorder component and (*b*) the minor component. The hydrogen bonds (see Table 1 ▸) are shown as dashed lines.

A view along the *a* axis of the crystal packing of CNBA is presented in Fig. 4 ▸, and details of the hydrogen bonding are given in Table 1 ▸. The crystal packing of the individual components, CBNA_1 and CBNA_2, are given in Fig. 5 ▸ *a* and 5*b*, respectively. In Fig. 5 ▸ *a* it can be seen that the mol­ecular packing for CNBA_1 is influenced by two C—H⋯O inter­actions: namely, C5—H5⋯O2 and C13—H13⋯O1. The first of these links the mol­ecules into *C*(11) chains and the second generates *C*(6) chains. In Fig. 5 ▸ *b*, it can be seen that for CNBA_2 the mol­ecular packing features weak C—H⋯Cl inter­actions (Table 1 ▸). As a result of these inter­actions, corrugated layers are formed, lying parallel to the *ac* plane.

## Database survey   

A search of the Cambridge Structural Database (Version 5.41, last update November 2019; Groom *et al.*, 2016 ▸) for *N*,1-di­phenyl­methanimines gave 73 hits for 63 compounds, while a search for 1-(4-nitro­phen­yl)-*N*-phenyl­methanimines gave 25 hits for six compounds. In these searches a number of compounds have multiple reports, or have been studied at different temperatures, or concern polymorphs.

The most relevant compounds that concern us here include those reported above in §1 (*Chemical context*), *viz. N*-(4-nitro­benzyl­idene)aniline (CSD refcodes QQQAIY01, QQQAIY02, QQQAIY03: Harada *et al.*, 2004*b* ▸), the 4-fluoro derivative (MIMDUJ: Subashini *et al.*, 2013*b* ▸), the 4-bromo derivative (FIBXIZ01: Subashini *et al.*, 2013*a* ▸), the 4-methyl derivative (NMBYAN: Filipenko *et al.*, 1976 ▸; NMBYAN22: Filipenko *et al.*, 1977 ▸; NMBYAN01: Cole *et al.*, 2001 ▸; NMBYAN25, NMBYAN26: Harada *et al.*, 2004*a* ▸), the 4-meth­oxy benzyl­idene derivative (NMBZYA01, NMBZYA02: Harada *et al.*, 2004*a* ▸) and the 4-hy­droxy derivative (WOTQED: Atioğlu *et al.*, 2015 ▸).

For *N*-(4-nitro­benzyl­idene)aniline measured at 300 K (QQQAIY01), one of the two independent mol­ecules in the asymmetric unit has type *D*1 disorder. At 200 K (QQQAIY02) a difference-Fourier map indicated only a few weak residual density peaks corresponding to the minor component, while at 90 K (QQQAIY03) no disorder was observed. For the 4-fluoro derivative measured at 173 K (MIMDUJ) no disorder was observed. For the 4-bromo derivative (FIBXIZ01), the crystals were incommensurate and twinned and the structure was refined in space group *A*2. A triclinic polymorph of the 4-methyl derivative (NMBYAN) with two independent mol­ecules in the asymmetric unit was reported on by Filipenko *et al.* (1976 ▸). A monoclinic polymorph, with one mol­ecule in the asymmetric unit, was reported on first by Filipenko *et al.* (1976 ▸) for NMBYAN22, and later a neutron diffraction study at 20 K was carried out by Cole *et al.* (2001 ▸) for NMBYAN01. The triclinic polymorph was also studied by Harada *et al.* (2004*a* ▸), at 300 K (NMBYAN25) and at 90 K (NMBYAN26) and showed only disorder of the methyl hydrogen atoms at both temperatures. The 4-meth­oxy benzyl­idene derivative, measured at 300 K (NMBZYA01) and 90 K (NMBZYA02), showed no disorder at either temperature. Finally, the 4-hy­droxy derivative, WOTQED, crystallizes with four independent mol­ecules in the asymmetric unit, and one of the mol­ecules has type *D*1 disorder.

The N=C bond lengths vary from as short as *ca*.1.187 Å, in one of the four independent mol­ecules of WOTQED, to *ca* 1.281 Å in NMBZYA02. In the title compound, the N1=C7 bond length in the major component is 1.291 (6) Å, while for the minor component the N21=C27 bond length is 1.234 (12) Å. In the above-mentioned compounds, the benzene rings are inclined to each other by dihedral angles varying from *ca* 2.24° in one of the independent mol­ecules of WOTQED to *ca* 55.76° in one of the two independent mol­ecules in NMBYAN26; thus the dihedral angles for the disorder components of the title compound fall roughly in the middle of this range.

## Hirshfeld surface analysis and two-dimensional fingerprint plots   

The Hirshfeld surface analysis (Spackman & Jayatilaka, 2009 ▸) and the associated two-dimensional fingerprint plots (McKinnon *et al.*, 2007 ▸) were performed with *CrystalExplorer17* (Turner *et al.*, 2017 ▸) following the protocol of Tiekink and collaborators (Tan *et al.*, 2019 ▸).

**Figure 6:**
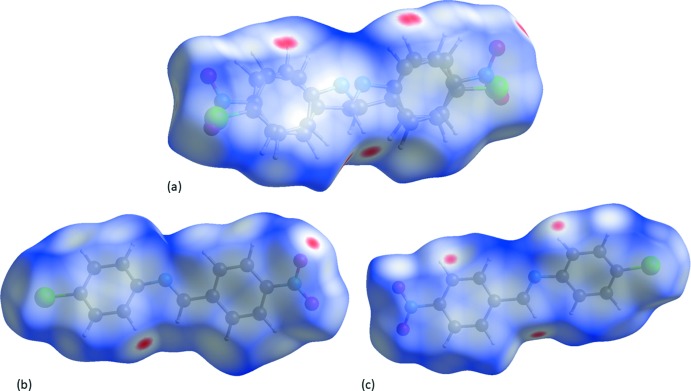
(*a*) The Hirshfeld surface of CNBA mapped over *d*_norm_, in the colour range −0.15 to 1.13 a.u., (*b*) the Hirshfeld surface of CNBA_1 mapped over *d*_norm_, in the colour range −0.14 to 1.32 a.u., (*c*) the Hirshfeld surface of CNBA_2 mapped over *d*_norm_, in the colour range −0.15 to 1.29 a.u.

The Hirshfeld surface of CNBA mapped over *d*_norm_ is given in Fig. 6 ▸ *a*, where short inter­atomic contacts are indicated by the red spots. The Hirshfeld surfaces of the individual components, CNBA_1 and CNBA_2, mapped over *d*_norm_ are given in Fig. 6 ▸ *b* and 6*c*, respectively.

**Figure 7:**
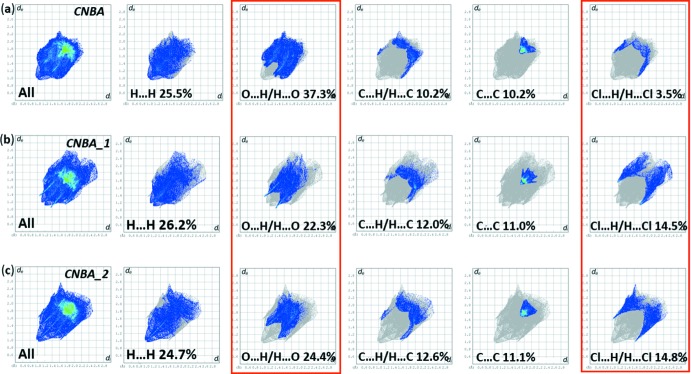
(*a*) The full two-dimensional fingerprint plot for CNBA, and fingerprint plots delineated into H⋯H, O⋯H/H⋯O, C⋯H/H⋯C, C⋯C and Cl⋯H/H⋯Cl contacts, (*b*) the full two-dimensional fingerprint plot for CNBA_1, and fingerprint plots delineated into H⋯H, O⋯H/H⋯O, C⋯H/H⋯C, C⋯C and Cl⋯H/H⋯Cl contacts, (*c*) the full two-dimensional fingerprint plot for CNBA_2, and fingerprint plots delineated into H⋯H, O⋯H/H⋯O, C⋯H/H⋯C, C⋯C and Cl⋯H/H⋯Cl contacts.

The full two-dimensional fingerprint plots for CNBA and for the individual components, CNBA_1 and CNBA_2, are given in Fig. 7 ▸ *a*, 7*b* and 7*c*, respectively. The relative percentage contributions of close contacts to the Hirshfeld surface for CNBA and for the individual components are compared in Table 2 ▸. For CNBA the principal inter­molecular inter­actions are delineated into O⋯H/H⋯O at 37.3%, H⋯H at 25.5%, C⋯H/H⋯C and C⋯C both at 10.2%, followed by Cl⋯H/H⋯Cl and N⋯H/H⋯N contacts both at 3.5%. For CNBA_1 and CMBA_2 the order is somewhat different with H⋯H contributions being superior or almost equal to the contributions of the O⋯H/H⋯O contacts, the latter contributions being 22.3 and 24.4%, respectively, compared to 37.3% for CNBA. In contrast, the Cl⋯H/H⋯Cl contacts contribute 14.5% and 14.8% for CNBA_1 and CNBA_2, respectively, compared to only 3.5% for CNBA. This situation reflects the details of the hydrogen bonding in the crystal structure (see Fig. 5 ▸ *a* and 5*b*, and Table 1 ▸).

## Synthesis and crystallization   

The commercially available organic compounds *p*-nitro­benzaldehyde and *p*-chloro­aniline were used without further purification and the title compound was synthesized following reported procedures (Batra *et al.*, 2004 ▸; Subashini *et al.*, 2013*a* ▸): the two reactants were taken in equimolar ratio and refluxed in ethanol for 6 h. On cooling, the synthesized compound was deposited at room temperature as a deep-yellow microcrystalline powder. The material was purified by repeated recrystallization using ethanol at room temperature and the purity of the sample was confirmed by thin layer chromatography. A saturated solution of CNBA was prepared using mixed solvents of ethanol and ethyl­acetate (1:1, *v*:*v*) and single crystals were obtained as yellow rods by slow evaporation of the solvents at room temperature over a period of 18 days. The ^1^H NMR spectrum of CNBA recorded in CDCl_3_ is shown in the supporting information, Fig. 1S, and the FTIR and FT Raman spectra are shown in Fig. 2S.

## Refinement   

Crystal data, data collection and structure refinement details are summarized in Table 3 ▸. The C-bound H atoms were included in calculated positions and refined as riding: C—H = 0.95 Å with *U*_iso_(H) = 1.2*U*_eq_(C). The mol­ecule is disordered with an occupancy ratio that after refinement was fixed at 0.649: 0.351. The benzene rings in the two components were refined as rigid bodies and the anisotropic displacement parameters of corresponding C atoms were made equal.

## Supplementary Material

Crystal structure: contains datablock(s) I, Global. DOI: 10.1107/S2056989020002212/hb7888sup1.cif

Structure factors: contains datablock(s) I. DOI: 10.1107/S2056989020002212/hb7888Isup2.hkl

NMR spectra. DOI: 10.1107/S2056989020002212/hb7888sup3.tif

FTIR and FT Raaman spectra. DOI: 10.1107/S2056989020002212/hb7888sup4.tif

Supporting information file. DOI: 10.1107/S2056989020002212/hb7888Isup5.cml

CCDC reference: 941508

Additional supporting information:  crystallographic information; 3D view; checkCIF report

## Figures and Tables

**Table 1:** Hydrogen-bond geometry (Å, °)

*D*—H⋯*A*	*D*—H	H⋯*A*	*D*⋯*A*	*D*—H⋯*A*
C5—H5⋯O2^i^	0.95	2.55	3.489 (7)	172
C13—H13⋯O1^ii^	0.95	2.57	3.426 (5)	151
C27—H27⋯Cl2^i^	0.95	2.78	3.661 (11)	155

Symmetry codes: (i) 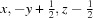; (ii) 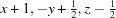.

**Table 2:** Percentage contributions of inter­atomic contacts to the Hirshfeld surface for CNBA, and for the individual disordered components, CNBA_1 and CNBA_2

Contact	Percentage contributions		
	CNBA	CNBA_1	CNBA_2
H⋯H	25.5	26.2	24.7
O⋯H/H⋯O	37.3	22.3	24.4
Cl⋯H/H⋯Cl	3.5	14.5	14.8
C⋯H/H⋯C	10.2	12.0	12.6
C⋯C	10.2	11.0	11.1
N⋯H/H⋯N	3.5	4.2	3.4
C⋯N	3.2	2.4	2.4
O⋯O	2.3	0.3	0.6
C⋯O	1.6	1.9	0.7
O⋯Cl	0.8	2.0	1.4
C⋯Cl	0.4	1.0	1.9

**Table 3:** Experimental details

Crystal data	
Chemical formula	C_13_H_9_ClN_2_O_2_
*M*_r_	260.67
Crystal system, space group	Monoclinic, *P*2_1_/*c*
Temperature (K)	173
*a*, *b*, *c* (Å)	3.8195 (5), 22.826 (4), 13.6381 (19)
β (°)	92.829 (11)
*V* (Å^3^)	1187.6 (3)
*Z*	4
Radiation type	Mo *K*α
μ (mm^−1^)	0.32
Crystal size (mm)	0.34 × 0.17 × 0.09

Data collection	
Diffractometer	STOE IPDS2
Absorption correction	Multi-scan (*MULABS*; Spek, 2020 ▸)
*T*_min_, *T*_max_	0.923, 1.000
No. of measured, independent and observed [*I* > 2σ(*I*)] reflections	9572, 2256, 1498
*R*_int_	0.046
(sin θ/λ)_max_ (Å^−1^)	0.610

Refinement	
*R*[*F* ^2^ > 2σ(*F* ^2^)], *wR*(*F* ^2^), *S*	0.049, 0.121, 0.98
No. of reflections	2256
No. of parameters	206
H-atom treatment	H-atom parameters constrained
Δρ_max_, Δρ_min_ (e Å^−3^)	0.30, −0.19

Computer programs: *X-AREA* and *X-RED32* (Stoe & Cie, 2006 ▸), *SHELXS97* (Sheldrick, 2008 ▸), *SHELXL2018* (Sheldrick, 2015 ▸), *PLATON* (Spek, 2020 ▸), *Mercury* (Macrae *et al.*, 2020 ▸) and *publCIF* (Westrip, 2010 ▸).

## supplementary crystallographic information

### Crystal data

**Table:**

C_13_H_9_ClN_2_O_2_	*F*(000) = 536
*M**_r_* = 260.67	*D*_x_ = 1.458 Mg m^−^^3^
Monoclinic, *P*2_1_/*c*	Mo *K*α radiation, λ = 0.71073 Å
*a* = 3.8195 (5) Å	Cell parameters from 3565 reflections
*b* = 22.826 (4) Å	θ = 1.7–26.0°
*c* = 13.6381 (19) Å	µ = 0.32 mm^−^^1^
β = 92.829 (11)°	*T* = 173 K
*V* = 1187.6 (3) Å^3^	Rod, yellow
*Z* = 4	0.34 × 0.17 × 0.09 mm

### Data collection

**Table:**

STOE IPDS-2 diffractometer	2256 independent reflections
Radiation source: fine-focus sealed tube	1498 reflections with *I* > 2σ(*I*)
Graphite monochromator	*R*_int_ = 0.046
phi & ω scans	θ_max_ = 25.7°, θ_min_ = 1.7°
Absorption correction: multi-scan (MULABS; Spek, 2020)	*h* = −4→4
*T*_min_ = 0.923, *T*_max_ = 1.000	*k* = −27→27
9572 measured reflections	*l* = −16→16

### Refinement

**Table:**

Refinement on *F*^2^	Secondary atom site location: difference Fourier map
Least-squares matrix: full	Hydrogen site location: inferred from neighbouring sites
*R*[*F*^2^ > 2σ(*F*^2^)] = 0.049	H-atom parameters constrained
*wR*(*F*^2^) = 0.121	*w* = 1/[σ^2^(*F*_o_^2^) + (0.0668*P*)^2^] where *P* = (*F*_o_^2^ + 2*F*_c_^2^)/3
*S* = 0.98	(Δ/σ)_max_ < 0.001
2256 reflections	Δρ_max_ = 0.30 e Å^−^^3^
206 parameters	Δρ_min_ = −0.19 e Å^−^^3^
0 restraints	Extinction correction: (SHELXL2018; Sheldrick, 2015), Fc^*^=kFc[1+0.001xFc^2^λ^3^/sin(2θ)]^-1/4^
Primary atom site location: structure-invariant direct methods	Extinction coefficient: 0.036 (4)

### Special details

**Table:**

Geometry. All esds (except the esd in the dihedral angle between two l.s. planes) are estimated using the full covariance matrix. The cell esds are taken into account individually in the estimation of esds in distances, angles and torsion angles; correlations between esds in cell parameters are only used when they are defined by crystal symmetry. An approximate (isotropic) treatment of cell esds is used for estimating esds involving l.s. planes.

### Fractional atomic coordinates and isotropic or equivalent isotropic displacement parameters (Å^2^)

**Table:**

	*x*	*y*	*z*	*U*_iso_*/*U*_eq_	Occ. (<1)
Cl1	1.0727 (7)	0.60002 (12)	0.0780 (3)	0.0575 (7)	0.649
O1	0.0813 (9)	0.23891 (13)	0.7308 (2)	0.0628 (8)	0.649
O2	0.212 (2)	0.1651 (2)	0.6402 (7)	0.069 (2)	0.649
N1	0.7162 (7)	0.42998 (14)	0.3862 (3)	0.0326 (7)	0.649
N2	0.1963 (14)	0.2188 (2)	0.6565 (4)	0.0444 (13)	0.649
C1	0.9720 (11)	0.54801 (15)	0.16579 (17)	0.0369 (11)	0.649
C2	1.0661 (9)	0.55941 (11)	0.26368 (19)	0.0379 (10)	0.649
H2	1.184626	0.594746	0.281349	0.045*	0.649
C3	0.9869 (8)	0.51912 (12)	0.33572 (13)	0.0334 (8)	0.649
H3	1.051256	0.526918	0.402629	0.040*	0.649
C4	0.8136 (8)	0.46743 (11)	0.30987 (16)	0.0297 (8)	0.649
C5	0.7194 (11)	0.45602 (14)	0.21198 (19)	0.0353 (10)	0.649
H5	0.600963	0.420689	0.194313	0.042*	0.649
C6	0.7987 (12)	0.49631 (18)	0.13994 (13)	0.0391 (11)	0.649
H6	0.734329	0.488517	0.073031	0.047*	0.649
C7	0.7008 (13)	0.3742 (2)	0.3713 (5)	0.0335 (11)	0.649
H7	0.774112	0.358824	0.310888	0.040*	0.649
C8	0.5727 (10)	0.33367 (11)	0.44542 (19)	0.0305 (10)	0.649
C9	0.4512 (9)	0.35627 (9)	0.5321 (2)	0.0346 (9)	0.649
H9	0.451447	0.397391	0.542816	0.042*	0.649
C10	0.3292 (9)	0.31870 (11)	0.60298 (18)	0.0337 (9)	0.649
H10	0.246154	0.334147	0.662194	0.040*	0.649
C11	0.3289 (11)	0.25854 (11)	0.5872 (2)	0.0278 (9)	0.649
C12	0.4504 (13)	0.23594 (9)	0.5006 (3)	0.0331 (10)	0.649
H12	0.450148	0.194817	0.489855	0.040*	0.649
C13	0.5724 (12)	0.27351 (12)	0.4297 (2)	0.0333 (10)	0.649
H13	0.655443	0.258061	0.370476	0.040*	0.649
Cl2	0.1792 (12)	0.1871 (2)	0.6481 (4)	0.0553 (12)	0.351
O21	1.1934 (18)	0.6433 (2)	0.1833 (4)	0.0704 (17)	0.351
O22	1.091 (5)	0.5975 (7)	0.0487 (13)	0.099 (8)	0.351
N21	0.6719 (15)	0.3947 (3)	0.4304 (6)	0.0429 (14)	0.351
N22	1.095 (2)	0.6024 (4)	0.1364 (8)	0.053 (2)	0.351
C21	0.331 (3)	0.2457 (2)	0.5774 (7)	0.0369 (11)	0.351
C22	0.476 (3)	0.2384 (3)	0.4867 (7)	0.0379 (10)	0.351
H22	0.496956	0.200313	0.459614	0.045*	0.351
C23	0.590 (3)	0.2869 (4)	0.4356 (5)	0.0334 (8)	0.351
H23	0.689079	0.281974	0.373553	0.040*	0.351
C24	0.559 (2)	0.3427 (3)	0.4752 (5)	0.0297 (8)	0.351
C25	0.414 (2)	0.3500 (2)	0.5659 (5)	0.0353 (10)	0.351
H25	0.393402	0.388076	0.592940	0.042*	0.351
C26	0.300 (2)	0.3015 (3)	0.6170 (4)	0.0391 (11)	0.351
H26	0.201277	0.306416	0.679004	0.047*	0.351
C27	0.652 (2)	0.3978 (5)	0.3399 (7)	0.039 (2)	0.351
H27	0.556380	0.365770	0.303238	0.047*	0.351
C28	0.7743 (17)	0.4503 (2)	0.2864 (5)	0.0305 (10)	0.351
C29	0.9255 (16)	0.4963 (3)	0.3401 (3)	0.0346 (9)	0.351
H29	0.953565	0.493632	0.409583	0.042*	0.351
C30	1.0355 (18)	0.5463 (2)	0.2922 (5)	0.0337 (9)	0.351
H30	1.138834	0.577751	0.328889	0.040*	0.351
C31	0.994 (2)	0.5502 (3)	0.1906 (5)	0.0278 (9)	0.351
C32	0.843 (2)	0.5042 (4)	0.1369 (3)	0.0331 (10)	0.351
H32	0.815122	0.506868	0.067426	0.040*	0.351
C33	0.733 (2)	0.4542 (3)	0.1848 (5)	0.0333 (10)	0.351
H33	0.629851	0.422748	0.148118	0.040*	0.351

### Atomic displacement parameters (Å^2^)

**Table:**

	*U*^11^	*U*^22^	*U*^33^	*U*^12^	*U*^13^	*U*^23^
Cl1	0.0600 (10)	0.0436 (10)	0.070 (2)	0.0011 (8)	0.0144 (12)	0.0180 (13)
O1	0.080 (2)	0.070 (2)	0.0402 (16)	−0.0056 (16)	0.0215 (16)	0.0067 (15)
O2	0.086 (4)	0.044 (4)	0.078 (3)	0.004 (3)	0.019 (3)	0.017 (3)
N1	0.0346 (17)	0.0294 (16)	0.0340 (17)	−0.0007 (13)	0.0049 (13)	0.0029 (17)
N2	0.039 (2)	0.048 (3)	0.046 (2)	0.005 (3)	−0.0023 (16)	0.014 (3)
C1	0.038 (2)	0.0319 (18)	0.040 (2)	0.0038 (16)	0.000 (2)	−0.0006 (18)
C2	0.039 (2)	0.0315 (18)	0.044 (3)	0.0009 (15)	0.0093 (17)	0.0016 (15)
C3	0.0362 (19)	0.0280 (18)	0.0363 (19)	−0.0026 (14)	0.0043 (15)	0.0024 (14)
C4	0.0270 (17)	0.0295 (18)	0.0327 (19)	0.0002 (14)	0.0011 (15)	−0.0044 (17)
C5	0.0339 (19)	0.0328 (19)	0.039 (3)	−0.0006 (14)	0.0036 (18)	0.0001 (18)
C6	0.036 (2)	0.039 (2)	0.043 (2)	−0.0028 (17)	0.0006 (16)	0.0048 (17)
C7	0.029 (2)	0.037 (3)	0.034 (3)	0.005 (2)	0.0000 (19)	−0.001 (2)
C8	0.0267 (16)	0.035 (2)	0.0289 (19)	−0.0004 (14)	−0.0039 (16)	−0.0050 (16)
C9	0.0364 (19)	0.0323 (17)	0.035 (2)	−0.0008 (14)	0.0005 (17)	0.0035 (15)
C10	0.0367 (19)	0.0319 (19)	0.0320 (18)	0.0049 (16)	−0.0027 (15)	−0.0071 (16)
C11	0.0271 (19)	0.029 (2)	0.0267 (17)	0.0014 (15)	−0.0009 (14)	0.0031 (16)
C12	0.032 (2)	0.0383 (19)	0.030 (2)	0.0029 (15)	0.0022 (16)	0.0033 (15)
C13	0.0344 (19)	0.033 (2)	0.0329 (18)	0.0014 (17)	0.0026 (15)	0.0015 (15)
Cl2	0.0509 (15)	0.057 (4)	0.058 (2)	0.007 (3)	0.0044 (13)	0.023 (3)
O21	0.101 (5)	0.041 (3)	0.069 (4)	−0.019 (3)	0.007 (3)	−0.008 (3)
O22	0.153 (13)	0.064 (7)	0.079 (12)	−0.017 (6)	−0.015 (8)	−0.009 (6)
N21	0.036 (3)	0.040 (4)	0.053 (4)	0.002 (3)	0.001 (3)	−0.004 (4)
N22	0.045 (4)	0.052 (5)	0.063 (6)	−0.014 (3)	0.010 (5)	−0.018 (5)
C21	0.038 (2)	0.0319 (18)	0.040 (2)	0.0038 (16)	0.000 (2)	−0.0006 (18)
C22	0.039 (2)	0.0315 (18)	0.044 (3)	0.0009 (15)	0.0093 (17)	0.0016 (15)
C23	0.0362 (19)	0.0280 (18)	0.0363 (19)	−0.0026 (14)	0.0043 (15)	0.0024 (14)
C24	0.0270 (17)	0.0295 (18)	0.0327 (19)	0.0002 (14)	0.0011 (15)	−0.0044 (17)
C25	0.0339 (19)	0.0328 (19)	0.039 (3)	−0.0006 (14)	0.0036 (18)	0.0001 (18)
C26	0.036 (2)	0.039 (2)	0.043 (2)	−0.0028 (17)	0.0006 (16)	0.0048 (17)
C27	0.029 (4)	0.041 (6)	0.048 (6)	0.003 (4)	0.001 (4)	−0.010 (5)
C28	0.0267 (16)	0.035 (2)	0.0289 (19)	−0.0004 (14)	−0.0039 (16)	−0.0050 (16)
C29	0.0364 (19)	0.0323 (17)	0.035 (2)	−0.0008 (14)	0.0005 (17)	0.0035 (15)
C30	0.0367 (19)	0.0319 (19)	0.0320 (18)	0.0049 (16)	−0.0027 (15)	−0.0071 (16)
C31	0.0271 (19)	0.029 (2)	0.0267 (17)	0.0014 (15)	−0.0009 (14)	0.0031 (16)
C32	0.032 (2)	0.0383 (19)	0.030 (2)	0.0029 (15)	0.0022 (16)	0.0033 (15)
C33	0.0344 (19)	0.033 (2)	0.0329 (18)	0.0014 (17)	0.0026 (15)	0.0015 (15)

### Geometric parameters (Å, º)

**Table:**

Cl1—C1	1.743 (4)	Cl2—C21	1.763 (7)
O1—N2	1.214 (6)	O21—N22	1.182 (9)
O2—N2	1.248 (7)	O22—N22	1.20 (2)
N1—C7	1.291 (6)	N21—C27	1.234 (12)
N1—C4	1.411 (4)	N21—C24	1.412 (9)
N2—C11	1.420 (6)	N22—C31	1.462 (11)
C1—C2	1.3900	C21—C22	1.3900
C1—C6	1.3900	C21—C26	1.3900
C2—C3	1.3900	C22—C23	1.3900
C2—H2	0.9500	C22—H22	0.9500
C3—C4	1.3900	C23—C24	1.3900
C3—H3	0.9500	C23—H23	0.9500
C4—C5	1.3900	C24—C25	1.3900
C5—C6	1.3900	C25—C26	1.3900
C5—H5	0.9500	C25—H25	0.9500
C6—H6	0.9500	C26—H26	0.9500
C7—C8	1.471 (6)	C27—C28	1.488 (11)
C7—H7	0.9500	C27—H27	0.9500
C8—C9	1.3900	C28—C29	1.3900
C8—C13	1.3900	C28—C33	1.3900
C9—C10	1.3900	C29—C30	1.3900
C9—H9	0.9500	C29—H29	0.9500
C10—C11	1.3900	C30—C31	1.3900
C10—H10	0.9500	C30—H30	0.9500
C11—C12	1.3900	C31—C32	1.3900
C12—C13	1.3900	C32—C33	1.3900
C12—H12	0.9500	C32—H32	0.9500
C13—H13	0.9500	C33—H33	0.9500
			
C7—N1—C4	119.5 (4)	C27—N21—C24	118.5 (9)
O1—N2—O2	122.8 (8)	O21—N22—O22	126.8 (14)
O1—N2—C11	117.9 (4)	O21—N22—C31	117.0 (9)
O2—N2—C11	119.2 (8)	O22—N22—C31	115.9 (12)
C2—C1—C6	120.0	C22—C21—C26	120.0
C2—C1—Cl1	118.5 (2)	C22—C21—Cl2	123.6 (5)
C6—C1—Cl1	121.5 (2)	C26—C21—Cl2	116.4 (5)
C3—C2—C1	120.0	C23—C22—C21	120.0
C3—C2—H2	120.0	C23—C22—H22	120.0
C1—C2—H2	120.0	C21—C22—H22	120.0
C2—C3—C4	120.0	C22—C23—C24	120.0
C2—C3—H3	120.0	C22—C23—H23	120.0
C4—C3—H3	120.0	C24—C23—H23	120.0
C5—C4—C3	120.0	C25—C24—C23	120.0
C5—C4—N1	122.0 (2)	C25—C24—N21	115.4 (6)
C3—C4—N1	117.8 (2)	C23—C24—N21	124.6 (6)
C4—C5—C6	120.0	C24—C25—C26	120.0
C4—C5—H5	120.0	C24—C25—H25	120.0
C6—C5—H5	120.0	C26—C25—H25	120.0
C5—C6—C1	120.0	C25—C26—C21	120.0
C5—C6—H6	120.0	C25—C26—H26	120.0
C1—C6—H6	120.0	C21—C26—H26	120.0
N1—C7—C8	121.7 (5)	N21—C27—C28	122.1 (10)
N1—C7—H7	119.1	N21—C27—H27	118.9
C8—C7—H7	119.1	C28—C27—H27	118.9
C9—C8—C13	120.0	C29—C28—C33	120.0
C9—C8—C7	119.2 (3)	C29—C28—C27	118.8 (6)
C13—C8—C7	120.8 (3)	C33—C28—C27	121.2 (6)
C8—C9—C10	120.0	C28—C29—C30	120.0
C8—C9—H9	120.0	C28—C29—H29	120.0
C10—C9—H9	120.0	C30—C29—H29	120.0
C11—C10—C9	120.0	C31—C30—C29	120.0
C11—C10—H10	120.0	C31—C30—H30	120.0
C9—C10—H10	120.0	C29—C30—H30	120.0
C10—C11—C12	120.0	C30—C31—C32	120.0
C10—C11—N2	121.8 (3)	C30—C31—N22	122.4 (7)
C12—C11—N2	118.2 (3)	C32—C31—N22	117.5 (7)
C13—C12—C11	120.0	C33—C32—C31	120.0
C13—C12—H12	120.0	C33—C32—H32	120.0
C11—C12—H12	120.0	C31—C32—H32	120.0
C12—C13—C8	120.0	C32—C33—C28	120.0
C12—C13—H13	120.0	C32—C33—H33	120.0
C8—C13—H13	120.0	C28—C33—H33	120.0
			
C6—C1—C2—C3	0.0	C26—C21—C22—C23	0.0
Cl1—C1—C2—C3	179.4 (3)	Cl2—C21—C22—C23	−179.9 (8)
C1—C2—C3—C4	0.0	C21—C22—C23—C24	0.0
C2—C3—C4—C5	0.0	C22—C23—C24—C25	0.0
C2—C3—C4—N1	−175.4 (3)	C22—C23—C24—N21	178.9 (8)
C7—N1—C4—C5	34.9 (4)	C27—N21—C24—C25	−147.7 (7)
C7—N1—C4—C3	−149.8 (4)	C27—N21—C24—C23	33.4 (10)
C3—C4—C5—C6	0.0	C23—C24—C25—C26	0.0
N1—C4—C5—C6	175.2 (3)	N21—C24—C25—C26	−179.0 (7)
C4—C5—C6—C1	0.0	C24—C25—C26—C21	0.0
C2—C1—C6—C5	0.0	C22—C21—C26—C25	0.0
Cl1—C1—C6—C5	−179.4 (3)	Cl2—C21—C26—C25	179.9 (7)
C4—N1—C7—C8	−174.8 (3)	C24—N21—C27—C28	−178.3 (6)
N1—C7—C8—C9	2.8 (6)	N21—C27—C28—C29	2.2 (11)
N1—C7—C8—C13	−177.2 (4)	N21—C27—C28—C33	−176.6 (7)
C13—C8—C9—C10	0.0	C33—C28—C29—C30	0.0
C7—C8—C9—C10	−180.0 (4)	C27—C28—C29—C30	−178.8 (7)
C8—C9—C10—C11	0.0	C28—C29—C30—C31	0.0
C9—C10—C11—C12	0.0	C29—C30—C31—C32	0.0
C9—C10—C11—N2	−178.0 (4)	C29—C30—C31—N22	178.0 (8)
O1—N2—C11—C10	0.1 (6)	O21—N22—C31—C30	−4.7 (12)
O2—N2—C11—C10	−177.9 (7)	O22—N22—C31—C30	170.1 (13)
O1—N2—C11—C12	−178.0 (4)	O21—N22—C31—C32	173.3 (7)
O2—N2—C11—C12	4.1 (9)	O22—N22—C31—C32	−11.8 (15)
C10—C11—C12—C13	0.0	C30—C31—C32—C33	0.0
N2—C11—C12—C13	178.1 (4)	N22—C31—C32—C33	−178.1 (8)
C11—C12—C13—C8	0.0	C31—C32—C33—C28	0.0
C9—C8—C13—C12	0.0	C29—C28—C33—C32	0.0
C7—C8—C13—C12	180.0 (4)	C27—C28—C33—C32	178.7 (7)

### Hydrogen-bond geometry (Å, º)

**Table:**

*D*—H···*A*	*D*—H	H···*A*	*D*···*A*	*D*—H···*A*
C5—H5···O2^i^	0.95	2.55	3.489 (7)	172
C13—H13···O1^ii^	0.95	2.57	3.426 (5)	151
C27—H27···Cl2^i^	0.95	2.78	3.661 (11)	155

Symmetry codes: (i) *x*, −*y*+1/2, *z*−1/2; (ii) *x*+1, −*y*+1/2, *z*−1/2.
