# Supplementary figures and images for: Whole-mol­ecule disorder of the Schiff base compound 4-chloro-N-(4-nitro­benzyl­idene)aniline: crystal structure and Hirshfeld surface analysis
Source: Acta Crystallogr E Crystallogr Commun. 2020 Feb 18;76(Pt 3):417–22. doi: 10.1107/S2056989020002212 (PMC7057374; doi:10.1107/S2056989020002212)

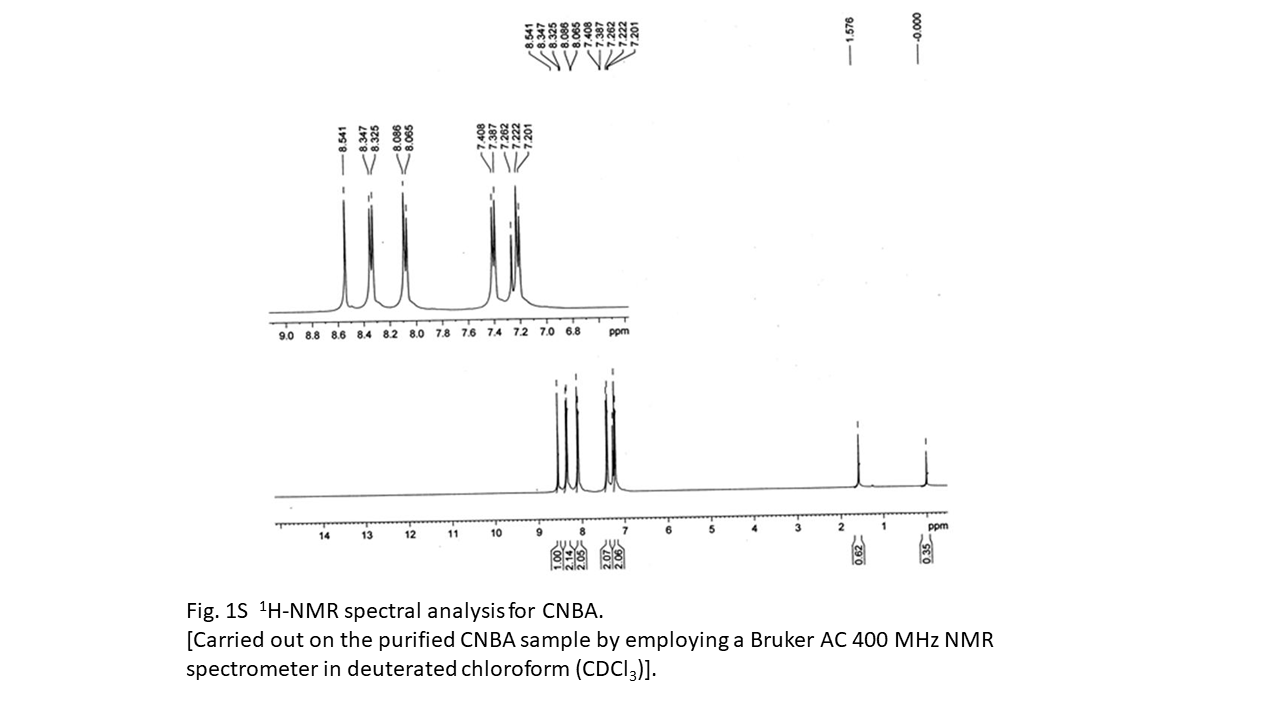

Supplement: Supplementary file 3 [file e-76-00417-sup3.tif]

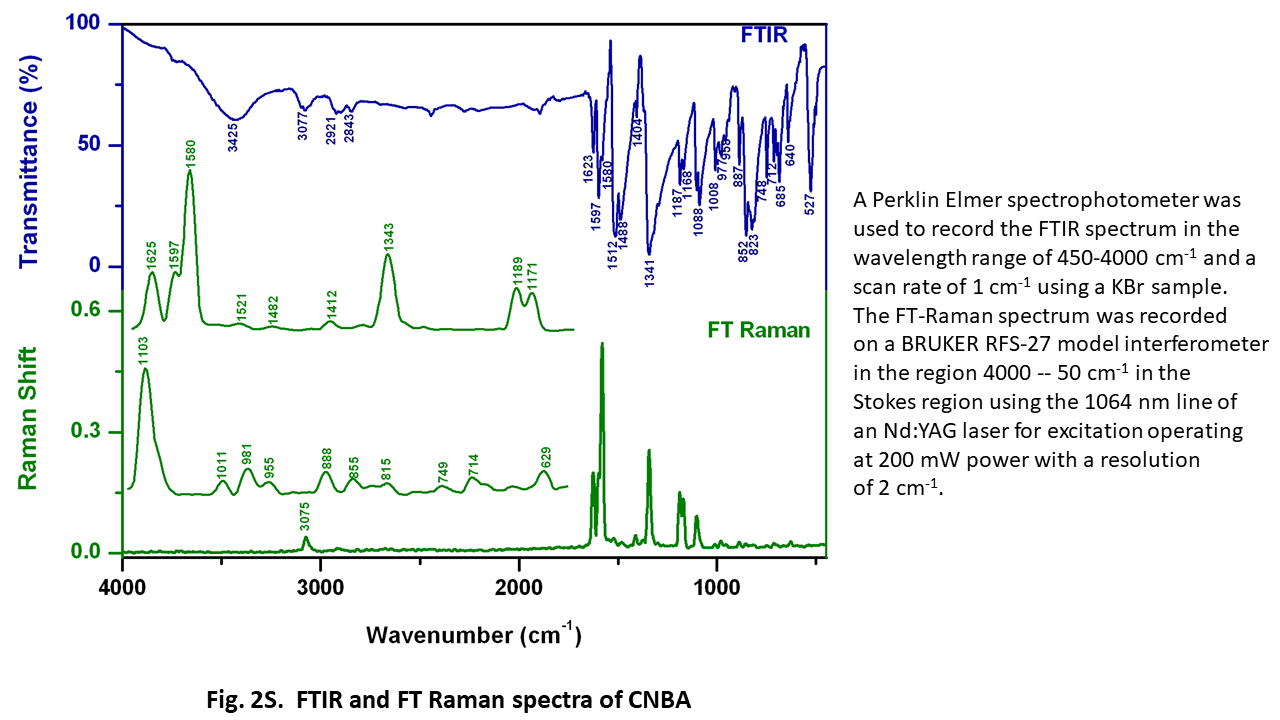

Supplement: Supplementary file 4 [file e-76-00417-sup4.tif]
